# A Reply to the Editor’s Query

**Published:** 1898-06

**Authors:** Wm. Ernest Walker

**Affiliations:** Pass Christian, Miss.


### Domestic Correspondence.

### A REPLY TO THE EDITOR'S QUERY.

To the Editor:

Sir,—I note on page 326 of your May issue a request for explanation of "Section 7, Article XIV.," Constitution of National Dental Association (the words quoted, however, being from Section 6, not 7). As Article XIV. is wholly devoted to the Branch feature of the National, my official position has caused me to study the provisions of this article very closely. I therefore venture my opinion as to the meaning of the section to which you refer; this, with the views of others which you will doubtless receive, will, I hope, help you to a clearer understanding. I should say that the words which you italicise—viz., "*And such delegates shall have the same standing in the National Association as though admitted* (*directly from the State societies*")—have the same significance as the following bracketed words, which I hope you will understand more readily: "And such delegates shall have the same standing in the National Association as though admitted (*at a meeting of the main body, instead of at a meeting of the Branch*)."

Yours truly,

WM. ERNEST WALKER.

PASS CHRISTIAN, MISS., May 9, 1898.

